# Effect of postpartum depression and role of infant feeding practices on relative weight of child at 1 and 3 years of age

**DOI:** 10.1186/s12884-024-06483-2

**Published:** 2024-05-02

**Authors:** Drishti Shrestha, Aliza K C Bhandari, Kohei Ogawa, Hisako Tanaka, Chiharu Miyayama, Reiko Horikawa, Kevin Y. Urayama, Naho Morisaki

**Affiliations:** 1 Graduate School of Public Health, St. Luke’s International University, Tokyo, Japan; 2 Department of Health Policy, National Center for Child Health and Development, Tokyo, Japan; 3 Division of Prevention, National Cancer Center Institute for Cancer Control, Tokyo, Japan; 4 Department of Social Medicine, National Center for Child Health and Development, Tokyo, Japan; 5 Center for Maternal-Fetal, Neonatal and Reproductive Medicine, National Center for Child Health and Development, Tokyo, Japan; 6 Department of Pediatrics, Juntendo University Faculty of Medicine, Tokyo, Japan; 7 Division of Endocrinology and Metabolism, National Center for Child Health and Development, Tokyo, Japan

**Keywords:** Postpartum, Maternal depression, Infant feeding practices, BMI, Japan

## Abstract

**Background:**

Childhood obesity has increased and is considered one of the most serious public health challenges of the 21^st^ century globally, and may be exacerbated by postpartum depression (PPD). The purpose of this study was to examine the association between PPD at 1^st^ and 6^th^ month postpartum, infant feeding practices, and body mass index (BMI) z-score of the child at one and three years of age.

**Methods:**

This study used data from an ongoing prospective maternal-child birth cohort performed at the National Center for Child Health and Development (NCCHD) in suburban Tokyo, Japan with the period of recruitment from May 13, 2010 to November 28, 2013. Out of 2,309 total number of mothers, 1,279 mother–child dyads were assessed in the study. We performed multivariable linear regression analysis to examine the association between PPD and child’s BMI z-score stratified by the child’s age at 1 year and 3 years of age.

**Results:**

The prevalence of PPD at 1 month postpartum (17%) was found to be higher than at 6 months (12%). In multivariable linear regression analysis we observed that children at 3 years who had mothers with PPD at 6 months had, on average, a BMI z-score 0.25 higher than children of mothers who did not have PPD at 6 months (ß coefficient 0.25, 95% CI [0.04 to 0.46], *p* value 0.02), holding all other covariates constant. Also, initiation of weaning food when child is at six months of age was associated with higher BMI z-score of the child at 3 years after adjusting for all covariates (ß coefficient = 0.18, 95% CI [0.03 to 0.34], *p*-value < 0.05).

**Conclusion:**

The significant association between PPD at 6 months and child’s BMI z-score at 3 years of age, in conjunction with birth trends and high prevalence of PPD, can add to the body of evidence that there is need for multiple assessment across the first postpartum year to rule out PPD as early screening and early interventions may benefit both maternal health and child development outcomes. These findings can indicate the need for establishing support systems for care-giving activities for mothers with PPD.

**Supplementary Information:**

The online version contains supplementary material available at 10.1186/s12884-024-06483-2.

## Background

Obesity is increasing at an alarming rate throughout the world. Childhood obesity is considered one of the most serious public health challenges of the 21^st^ century globally [1], and is associated with more deaths than childhood underweight conditions [2]. Since 1971, increasing trends have been reported in body mass index (BMI) among children in both high-income countries and low-income countries [3]. The global prevalence of overweight in children aged 5 years or under has increased modestly, along with increase in obesity in children aged 5 to 19 years between 1975 and 2016 [4]. According to the World Health Organization (WHO), the prevalence of overweight and obesity among children and adolescents aged 5 to 19 years has risen dramatically from 4% in 1975 to 18% in 2016 [5]. In Japan, the prevalence of overweight in children under 5 years is 1.5% which is less compared to that in the United States (9.4%) [6]. However, high prevalence of low birth weight (LBW) infants (9.5%) and increasing percentages of overweight/obese women aged 20 years or older (19.2%) in recent years [7] may have some lasting effects on the weight status in children of future generations in Japan.

Childhood obesity can lead to a course for an unhealthy adult life as it has been linked with a range of adverse physical and mental health outcomes. Previous studies have reported several factors associated with childhood overweight and/or obesity in children under 5 years of age such as maternal education, age at marriage, marital status, mother’s BMI, maternal depression, socio-economic status, child’s sex, birth weight, birth order, number of children, media exposure, high dietary diversity, etc. [8–11]. In addition, postpartum depression (PPD) is a common and serious mental health problem that is associated with maternal suffering and numerous negative consequences for the offspring [12, 13]. The prevalence of PPD has been reported to range from 10 to 15% in western countries to about 18% in the lower-middle income countries [14] with a prevalence of 13.7% in Japan [13]. PPD can compromise care-giving activities and performance of parenting roles including feeding practices such as breastfeeding, disruptions in sleep routines, decreases in post-natal health checkups and also decreases in vaccination rates among children [15], which may affect the child’s early development [16]. Attachment theory, and its application to parenting behavior, suggests that depression may influence caregiving through (a) reduced understanding of infants’ needs, (b) reduced sensitivity in responding to infant cues, and/or (c) reduced ability to comply with infant care guidance [17, 18]. Studies have shown that depressive symptoms are associated with reduced odds of continued breastfeeding, with increased practice of adding cereal to a bottle of formula or breastmilk, and higher bread/cereal and total energy intake at 2 months, but not associated with early introduction of complementary foods [19, 20].

A Brazilian cohort study showed a more rapid weight gain in children of mothers affected by PPD and suggested that disturbances in feeding behavior of depressed mothers could affect both tails of the growth distribution, leading either to stunting or obesity [11]. A study conducted in the United States (US) showed a small but significantly greater average weight gain at six months in infants of mothers with PPD, but PPD was not associated with infant feeding practices especially concerning early introduction of complementary foods [21]. There is a need to further understand the effect of PPD on weight status in early childhood and the role of feeding practices in influencing this relationship. Hence, our study aims to examine the association between maternal PPD at one and six months postpartum and the relative weight of a child measured by BMI z-score at age one and three years. The effect of infant feeding practices in influencing PPD and BMI z-score relationship will also be examined.

## Methods

### Study design

This study utilized data from an ongoing prospective birth cohort study performed at the National Center for Child Health and Development (NCCHD) in suburban Tokyo, Japan with the period of recruitment from May 13, 2010 to November 28, 2013. The pregnant women were recruited during their first antenatal visits, which usually took place during 6–14 weeks of gestation. During the first year of the postnatal period, follow-up was performed at one month, three months, and at 3-month intervals thereafter. The follow-up was conducted on an annual basis after the first year [22].

### Study population

**Fig. 1:**
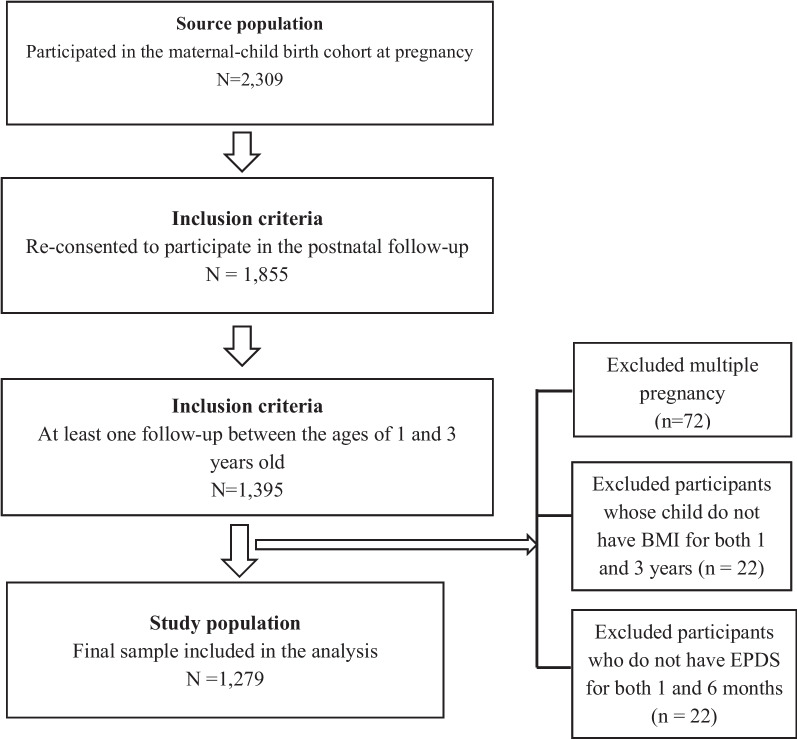
Flow diagram for study population

Of 2,309 women who participated in the birth cohort at pregnancy, we included 1,855 participants who re-consented at the postnatal follow-up and 1,395 participants had performed at least one follow-up of their child when they were one or three years of age. Among them, we excluded participants with multiple births (*n* = 72), participants whose child did not have BMI measures at both one and three years (*n* = 22) and participants who had missing information for Edinburgh Postnatal Depression Scale (EPDS) at both 1 and 6 months postpartum. Hence, the final number of participants included in the analysis was 1,279 mother–child dyads (Fig. 1).

### Data collection

Data were collected through self-administered questionnaires at recruitment, including information on basic sociodemographics, pregnancy characteristics and child characteristics. Information related to the pregnancy, delivery, and birth outcomes was obtained from the electronic medical records. Data collection at follow-up included a combination of self-administered questionnaires and requests for visits to the hospital for in-person anthropometric measurements by medical staff [23].

### Dependent variable

We used relative weight of child as the outcome variable, measured using BMI z-scores of children at one and three years of age. BMI z-score is a measure of weight (kg) adjusted for height (cm), age (months) and sex, relative to a smoothed reference distribution and represents the standard deviations from the mean of the population standard [24]. Thus, BMI z-score provides a numeric estimate of how far the measured value is from the average. In this study, the BMI z-score estimates were calculated using the Excel-based Clinical Tools for Growth Evaluation of Children sourced from The Japanese Society for Pediatric Endocrinology (JSPE) [25], which was created based on the “Infant Physical Growth Survey Report” published by the Ministry of Health, Labor and Welfare in 2000 and used as a continuous outcome [26, 27].

### Explanatory variables

Postpartum depression (PPD) was the primary exposure for this study, which is a major depressive disorder with a postpartum onset in the early months after childbirth. PPD was assessed using a Japanese version of the Edinburgh Postnatal Depression Scale (EPDS) at 1 month and 6 months postpartum. The EPDS is a 10-item self-report screening tool designed to screen women for symptoms of emotional distress during pregnancy and the postnatal period in which each item is scored on a 4-point scale ranging from 0 to 3 [28]. Total scores can range from 0 to 30, and the cut-off value of ≥ 9 has been shown to indicate good sensitivity and specificity for depression among Japanese women [29]. We derived a binary variable in which an EPDS score of ≥ 9 was defined as having symptoms of PPD. Since EPDS was only used to collect information and not as a clinical screening tool for PPD, no intervention was done among mothers who were identified to have PPD in this cohort.

In terms of assessment timing, childbirth is a difficult and exhausting process in which a female goes through a lot of hormonal, physical, emotional, and psychological changes throughout pregnancy and tremendous changes occur in the mother’s familial and interpersonal world which can lead to stress in earlier months of postpartum [30]. Two Japanese studies concluded the earlier months postpartum as risk factors for PPD showing the prevalence of PPD to be higher at 1-month postpartum than other times [31, 32]. Hence, we assessed PPD at two different points of time i.e. at 1-month and 6-months. In addition, we also assessed the proportion of mothers with PPD at 1-month only, 6-months only, PPD at both 1 and 6 months, and PPD at either 1 month or 6 months. In addition, we also assessed the information on sociodemographic characteristics of mothers stratified by PD.

Similarly, the information on exclusive breastfeeding (EBF) until 6 months and initiation of weaning food after 6 months was collected by a self-administered questionnaire during the follow-up period. We created a binary yes/no variable for both defined as practiced EBF vs did not practice EBF, and weaning started before or after six months vs at 6 months. We coded additional variables including maternal age (≤ 29, 30–34, 35–39, ≥ 40 years), maternal education (university, community college, training school, high school or less), marital status (single, married), annual household income (> 8 million yen, 4–8 million yen, < 4 million yen), occupation (unemployed, employed), pre-pregnancy BMI (18.5 to 25 kg/m^2^, < 18.5 kg/m^2^, ≥ 25 kg/m^2^) [27], complications during pregnancy (at least one complications such as gestational diabetes, pregnancy induced hypertension, preeclampsia, placenta accreta, placenta previa coded as yes and no), delivery method (vaginal delivery, cesarean section), primiparity (yes, no), preterm (gestational age at birth < 37 weeks, ≥ 37 weeks), and birth weight (categorization 1: < 2500gm, ≥ 2500gm and categorization 2: < 2500gm as low, 2500–4000 gm as normal and ≥ 4000gm as obese).

### Statistical analysis

The frequency and percentage of maternal characteristics, paternal characteristics, child characteristics and infant feeding practices were examined for the overall population. The mean with standard deviation of BMI z-score at 1 year and 3 years were assessed in relation to these characteristics. The student t-test or ANOVA were performed to assess the association between the explanatory variables and outcome.

We performed linear regression analysis to examine the association between PPD at 1 month and 6 months postpartum and BMI z-score of the child at ages 1 and 3 years. Hence, we pursued separate linear regression analyses to address four associations based on the timing of the maternal PPD and follow-up of the child at age 1 year and 3 years for outcome assessment. The model 1 represented a simple linear regression analysis examining the association between PPD at 1 or 6 months with child’s BMI z-score at 1 and 3 years of age separately. Model 2 included model 1 adjusted for sociodemographic variables of mother (maternal age, maternal education, marital status, income, occupation) and child characteristics (child gender, gestational age, birth weight) as potential confounders. Model 3 included model 2 adjusted for additional potential confounders (complications during pregnancy, delivery method, pre-pregnancy BMI, primiparity). Model 4 included model 3 adjusted for variables related to infant feeding practices (exclusive breastfeeding until 6 months and initiation of weaning food at 6 months). The prevalence of depression among women with multiple pregnancy has been repeatedly reported to be higher than those with singleton pregnancies hence, we dropped women with multiple pregnancy to avoid high-risk population [33]. Mediation analysis was considered based on the method by Baron and Kenny [34] to assess the hypothesis that PPD at 6 months postpartum would influence child’s BMI z-score at 3 years of age mediated by infant feeding practices like EBF and weaning. Infant feeding practices were also assessed for potential interaction with PPD at 6 months postpartum in influencing child’s BMI z-score at 3 years of age. For all statistical tests, a two-sided *p*-value of 0.05 was considered statistically significant. Statistical analysis were conducted using Stata BE 17.0.

### Ethical approval

Written informed consent was obtained from the participants to enroll into the original cohort study which was approved by the Institutional Review Board of the National Center for Child Health and Development. The data was obtained from the same institution upon the careful evaluation of the current study proposal.

## Results

Of the 1,279 eligible participants, 1,198 participants had information on BMI at 1 year of age and 914 at 3 years of age. Majority of the mothers (46.29%) were of age 35–39 years at delivery with mean age of 36.2 ± 4.12 years. Majority of the mothers (59.11%) had educational level up to university, were married (94.45%), and more than half were employed (53.79%) and had an annual household income of less than 4 million yen (53.17%). In addition, only about 5% of the mothers had a pre-pregnancy BMI of over 25 kg/m^2^ and 22.6% had complications during pregnancy. About 221 mothers (17%) had PPD at 1 month postpartum, 155 mothers (12%) had PPD at 6 months postpartum, 153 mothers (12.0%) had PPD at 1 months only, 87 mothers had PPD at 6 months only, 68 mothers (5.32%) had PPD at both 1 and 6 months and 186 mothers (14.54%) had PPD at either 1 or 6 months. When we stratified the study population by PPD, there were not much statistical difference in the population with PPD and without PPD except for their annual income and parity (Supplementary Table 1). More than half of mothers (56.92%) breastfed exclusively and started weaning at 6 months (53.24%). Based on crude analyses, the child’s BMI z-score was significantly associated with maternal age, pre-pregnancy BMI, birth weight, PPD at 6 months, EBF, and initiation of weaning food at 6 months during our univariable analysis (Table 1).

**Table 1:** Characteristics of the study population and relationship with child’s BMI z-score at 1 and 3 years of age

**Variables**	**Overall population (*N* = 1279)**	**Population with BMI z-score at 1 year (*N* = 1198)**			**Population with BMI z-score at 3 years (*N* = 914)**		
	**Frequency (%)**	**Frequency**	**Mean (sd)**	***P* value**	**Frequency**	**Mean (sd)**	***P* value**
**Maternal age (in years)**							
Mean (sd)	36.2 (4.12)						
≤ 29	88 (6.88)	83	-0.002 (0.88)	0.66	56	-0.16 (1.00)	0.001**
30–34	320 (25.02)	300	0.12 (0.82)		215	0.04 (0.92)	
35–39	592 (46.29)	553	0.13 (0.94)		423	0.26 (0.95)	
≥ 40	279 (21.81)	262	0.10 (0.92)		220	0.22 (0.93)	
**Maternal education**							
High school or less	75 (5.86)	73	-0.14 (0.90)	0.06	48	0.003 (1.08)	0.17
CT or VS or JC	387 (30.26)	366	0.12 (0.90)		281	0.12 (0.95)	
University	756 (59.11)	705	0.13 (0.90)		544	0.22 (0.93)	
Missing	61 (4.77)	54	0.19 (0.85)		41	0.17 (1.10)	
**Marital status**							
Single	11 (0.86)	11	0.01 (0.76)	0.72	7	-0.07 (0.63)	0.49
Married	1208 (94.45)	1133	0.11 (0.91)		867	0.17 (0.94)	
Missing	60 (4.69)	54	0.20(0.85)		40	0.19 (1.11)	
**Household annual income (in Japanese yen)**							
< 4 million	680 (53.17)	635	0.12 (0.88)	0.41	57	-0.02 (1.11)	0.10
4–8 million	400 (31.27)	382	0.14 (0.92)		290	0.14 (1.01)	
> 8 million	90 (7.04)	82	-0.001 (0.9)		490	0.23 (0.89)	
Missing	109 (8.52)	99	0.04 (0.96)		77	0.06 (0.93)	
**Occupation**							
Unemployed	527 (41.20)	495	0.12 (0.91)	0.65	367	0.10 (0.97)	0.04*
Employed	688 (53.79)	646	0.10 (0.90)		503	0.23 (0.92)	
Missing	64 (5.00)	57	0.20 (0.84)		44	0.17 (1.06)	
**Pre-pregnancy BMI (in kg/m**^**2**^**)**							
18.5–25	948 (74.12)	889	0.15 (0.87)	< 0.001***	676	0.21 (0.91)	< 0.001***
< 18.5	249 (19.47)	230	-0.14 (0.93)		179	-0.05 (0.98)	
≥ 25	68 (5.32)	66	0.47 (1.00)		50	0.50 (1.19)	
Missing	14 (1.09)	13	0.02 (1.10)		9	0.15 (0.66)	
**Complications during pregnancy **^**a**^** (at least one complications)**							
No	990 (77.4)	929	0.10 (0.90)	0.47	693	0.16 (0.93)	0.35
Yes	289 (22.6)	269	0.15 (0.93)		221	0.23 (1.02)	
**Delivery method**							
Vaginal delivery	878 (68.65)	827	0.10 (0.92)	0.56	616	0.18 (0.93)	0.97
CS section	364 (28.46)	338	0.14 (0.87)		276	0.18 (0.97)	
Missing	37 (2.89)	33	0.06 (0.86)		22	0.02 (1.17)	
**Primiparity**							
Yes	815 (63.72)	773	0.11 (0.87)	0.90	570	0.18 (0.95)	0.69
No	464 (36.28)	425	0.12 (0.96)		344	0.16 (0.94)	
**Paternal education**							
University	981 (76.70)	917	0.10 (0.91)	0.95	696	0.16 (0.94)	0.84
CT or VS Or JC	6 (0.47)	6	0.13 (0.81)		4	0.33 (0.44)	
High school or less	225 (17.59)	215	0.14 (0.90)		171	0.20 (0.96)	
Missing	67 (5.24)	60	0.20 (0.83)		43	0.19 (1.07)	
**Child’s sex**							
Female	630 (49.26)	588	0.14 (0.91)	0.21	441	0.13 (0.90)	0.20
Male	649 (50.74)	610	0.08 (0.89)		473	0.21 (0.99)	
**Gestational age (in weeks)**							
Preterm < 37 weeks	68 (5.32)	61	0.07 (0.90)	0.71	47	0.29 (1.19)	0.37
Fullterm ≥ 37 weeks	1210 (94.61)	1137	0.11 (0.90)		866	0.17 (0.94)	
Missing	1 (0.08)	0	0.00		1	-1.15	
**Birth weight (in grams)**							
Mean	2982.79						
Categorization 1 (< 2500, ≥ 2500)							
< 2500	131 (10.24)	122	-0.08 (0.83)	0.01*	90	-0.18 (1.06)	< 0.001***
≥ 2500	1148 (89.76)	1076	0.13 (0.91)		824	0.21 (0.93)	
Categorization 2 (< 2500, 2500—4000, ≥ 4000)							
< 2500 (Low)	131 (10.24)	122	-0.08 (0.83)	0.02*	90	-0.18 (1.06)	< 0.001***
2500–4000 (Normal)	1137 (88.90)	1065	0.13 (0.91)		817	0.21(0.93)	
≥ 4000 (Obese)	11 (0.86)	11	0.46 (0.73)		7	0.63 (0.63)	
**PPD at 1 month**							
No	942 (73.65)	877	0.12 (0.87)	0.24	668	0.15 (0.94)	0.19
Yes	221 (17.28)	208	0.05 (0.94)		157	0.25 (0.98)	
Missing	116 (9.07)	113	0.13 (1.04)		89	0.24 (0.997)	
**PPD at 6 months**							
No	945 (73.89)	901	0.10 (0.89)	0.54	672	0.14 (0.93)	0.04*
Yes	155 (12.12)	148	0.15 (0.96)		115	0.34 (1.04)	
Missing	179 (14)	149	0.14 (0.93)		127	0.20 (0.94)	
**PPD**							
PPD at 1 month only	153 (12.0)	144	0.04 (0.90)	0.30	102	0.20 (0.94)	0.50
PPD at 6 months only	87 (6.8)	84	0.22 (0.91)	0.33	60	0.32 (1.03)	0.16
PPD both at 1 and 6 months	68 (5.32)	64	0.07 (1.03)	0.66	55	0.36 (1.05)	0.18
PPD either at 1 month and 6 months	186 (14.54)	180	0.06 (0.90)		128	0.21 (0.98)	
No	730 (57.08)	692	0.12 (0.86)		515	0.13 (0.92)	
**Exclusive breastfeeding until 6 months**							
No	335 (26.19)	315	0.19 (0.87)	0.02*	246	0.30 (0.96)	0.001**
Yes	728 (56.92)	695	0.06 (0.90)		513	0.07 (0.91)	
Missing	216 (16.89)	188	0.19 (0.96)		155	0.32 (1.0)	
**Initiation of weaning food at 6 months**							
No	393 (30.73)	371	0.02 (0.92)	0.08	278	0.01 (0.93)	0.02*
Yes	681 (53.24)	651	0.13 (0.90)		487	0.22 (0.92)	
Missing	205 (16.03)	176	0.23 (0.85)		149	0.31 (1.03)	

*sd* Standard deviation, *PPD* Postpartum depression, *CT or VS or JC* College of technology or vocational school or junior college

^*^P-value < 0.05, ^**^p-value < 0.01, ^***^p value < 0.001

^a^Presence of at least one of the following complications: gestational diabetes mellitus, pregnancy induced hypertension, pre-eclampsia, placenta accreta and placenta previa

The results of multivariable linear regression model examining the association between PPD at 1 month and child’s BMI z-score at 1 year and 3 years are shown in Table 2. In the unadjusted analysis (Model 1), PPD at 1 month was not significantly associated with child’s BMI z-score at the age of 1 year (ß coefficient -0.08, 95% CI [0.21 to 0.05]) and at the age of 3 years (ß coefficient -0.11, 95% CI [-0.06 to 0.27]). However, the adjusted analysis additionally accounting for pregnancy characteristics (Model 3) showed a significant association between PPD at 1 month and BMI z-score at 3 years (ß coefficient 0.20, 95% CI [0.02 to 0.37]). Additionally adjusting for infant feeding practices (Model 4) slightly attenuated the association (ß coefficient 0.16, 95% CI [-0.04 to 0.36]).

**Table 2:** Multivariable linear regression model examining the association between PPD at 1 month and child's BMI z-score at the age of 1 year and 3 years

**Variables**	**Model 1**		**Model 2**		**Model 3**		**Model 4**	
	**ß coefficient (95% CI)**							
	**1 year (*N* = 1198)**	**3 years (*N* = 914)**	**1 year (*N* = 999)**	**3 years (*N* = 754)**	**1 year (*N* = 960)**	**3 years (*N* = 728)**	**1 year (*N* = 807)**	**3 years (*N* = 601)**
**PPD at 1 month (ref. No)**								
Yes	-0.08 (-0.21 to 0.05)	0.11 (-0.06 to 0.27)	-0.06 (-0.21 to 0.08)	0.14 (-0.03 to 0.32)	-0.02 (-0.17 to 0.13)	0.20* (0.02 to 0.37)	-0.05 (-0.22 to 0.12)	0.16 (-0.04 to 0.36)
**Maternal age (in years) (ref. ≤ 29)**								
30–34			0.07 (-0.16 to 0.33)	0.27 (-0.05 to 0.58)	0.003 (-0.25 to 0.25)	0.26 (-0.06 to 0.57)	-0.02 (-0.30 to 0.26)	0.16 (-0.19 to 0.53)
35–39			0.10 (-0.14 to 0.33)	0.50*** (0.20 to 0.80)	0.003 (-0.24 to 0.24)	0.45** (0.14 to 0.75)	(-0.03) (-0.30 to 0.24)	0.31 (-0.05 to 0.66)
≥ 40			0.07 (-0.18 to 0.32)	0.43* (0.12 to 0.74)	-0.03 (-0.28 to 0.23)	0.41* (0.09 to 0.73)	-0.10 (-0.39 to 0.19)	0.25 (-0.11 to 0.62)
**Maternal education (ref. high school or less)**								
CT or VS or JC			0.28* (0.03 to 0.53)	0.07 (-0.24 to 0.39)	0.33** (0.08 to 0.58)	0.10 (-0.22 to 0.42)	0.30* (0.02 to 0.58)	0.13 (-0.23 to 0.49)
University			0.29* (0.04 to 0.53)	0.20 (-0.11 to 0.51)	0.36** (0.11 to 0.60)	0.24 (-0.08 to 0.55)	0.31* (0.03 to 0.58)	0.22 (-0.13 to 0.58)
**Marital status (ref. single)**								
Married			0.04 (-0.53 to 0.60)	0.48 (-0.28 to 1.23)	0.01 (-0.58 to 0.60)	0.49 (-0.26 to 1.24)	-0.01 (-0.61 to 0.59)	0.36 (-0.39 to 1.11)
**Occupation (ref. unemployed)**								
Employed			0.01 (-0.11 to 0.12)	0.12 (-0.03 to 0.26)	0.01 (-0.11 to 0.13)	0.12 (-0.03 to 0.27)	0.03 (-0.10 to 0.17)	0.08 (-0.08 to 0.24)
**Income (ref. < 4 million yen)**								
4–8 million			0.06 (-0.17 to 0.29)	0.04 (-0.24 to 0.32)	0.09 (-0.14 to 0.32)	0.03 (-0.26 to 0.31)	0.05 ( -0.21 to 0.31)	-0.02 (-0.33 to 0.29)
> 8 million			0.03 (-0.19 to 0.26)	0.05 (-0.23 to 0.33)	0.07 (-0.16 to 0.30)	0.04 (-0.25 to 0.33)	0.02 (-0.24 to 0.27)	0.05 (-0.26 to 0.36)
**Child’s sex (ref. female)**								
Male			0.11 (-0.004 to 0.22)	0.004 (-0.13 to 0.14)	0.11 (-0.01 to 0.22)	0.01 (-0.13 to 0.14)	0.10 (-0.03 to 0.22)	0.02 (-0.13 to 0.17)
**Gestational week (ref. Preterm < 37 weeks)**								
Full term ≥ 37 weeks			-0.21 (-0.50 to 0.08)	-0.39 * (-0.73 to -0.04)	- 0.07 (-0.37 to 0.24)	-0.28 (-0.36 to 0.08)	-0.11 (-0.14 to 0.18)	-0.35 (-0.73 to 0.03)
**Birth weight (in grams) (ref. < 2500)**								
≥ 2500			0.30** (0.10 to 0.52)	0.50*** (0.25 to 0.75)	0.27* (0.05 to 0.48)	0.48*** (0.22 to 0.74)	0.26* (0.03 to 0.49)	0.39** (0.12 to 0.67)
**Complications (ref. No)**								
Yes					-0.01 (-0.15 to 0.15)	0.05 (-0.11 to 0.23)	0.02 (-0.14 to 0.18)	0.03 (-0.16 to 0.22)
**Delivery method (ref. Vaginal Delivery)**								
CS section					0.03 (-0.10 to 0.16)	-0.06 (-0.22 to 0.09)	0.0002 (-0.15 to 0.15)	-0.10 (-0.26 to 0.07)
**Pre pregnancy BMI (in kg/m**^**2**^**) (ref. 18.5 to 25)**								
< 18.5					-0.28*** (0.43 to -0.13)	-0.22* (-0.40 to -0.04)	-0.33*** (-0.50 to -0.17)	-0.30** (-0.50 to -0.11)
≥ 25					0.27* (0.20 to 0.52)	0.17 (-0.13 to 0.47)	0.23 (-0.04 to 0.49)	0.04 (-0.27 to 0.36)
**Primiparity (ref. Yes)**								
No					0.03 (-0.10 to 0.15)	0.05 (-0.09 to 0.20)	0.06 (-0.08 to 0.19)	0.01 (-0.15 to 0.18)
**Exclusive breastfeeding until 6 months (ref. No)**								
Yes							-0.16* (-0.30 to -0.02)	-0.19* (-0.36 to -0.03)
**Initiation of Weaning Food at 6 months (ref. No)**								
Yes							0.04 (-0.10 to 0.17)	0.15 (-0.01 to 0.31)

*CI* Confidence Interval, *BMI* Body Mass Index, *PPD* Postpartum depression, *CT or VS or JC* College of technology or vocational school or junior college

^*^P-value < 0.05, ^**^p-value < 0.01, ^***^p value < 0.001

The results of multivariable linear regression model examining the association between PPD at 6 months and child’s BMI z-score at 1 year and 3 years are shown in Table 3. In the unadjusted analysis (Model 1), PPD at 6 months was not significantly associated with child’s BMI z-score at the age of 1 year (ß coefficient 0.05, 95% CI [-0.11 to 0.21]) showing similar results for the adjusted analyses. However, a statistically significant association was observed for child’s BMI z-score at the age of 3 years in model 1 (ß coefficient 0.20, 95% CI [0.01 to 0.39]), and adjusted analysis did not considerably alter the results for BMI z-score at both 1 year and 3 years. Including infant feeding practices into the model showed a statistically significant independent effect of PPD at 6 months on child’s BMI z-score at the age of 3 years (ß coefficient 0.25, 95% CI [(0.04 to 0.46)]. Several covariates also appeared to be independently associated with BMI z-score at 3 years independent of the association for PPD at 6 months, including gestational age, birth weight, pre-pregnancy BMI, exclusive breastfeeding until 6 months and initiation of weaning food at 6 months.

**Table 3:** Multivariable linear regression model examining the association between PPD at 6 months and child's BMI z-score at the age of 1 year and 3 years

	**Model 1**		**Model 2**		**Model 3**		**Model 4**	
	**ß coefficient (95% CI)**							
	**1 year (*N* = 1198)**	**3 years (*N* = 914)**	**1 year (*N* = 956)**	**3 years (*N* = 713)**	**1 year (*N* = 918)**	**3 years (*N* = 689)**	**1 year (*N* = 800)**	**3 years (*N* = 600)**
**PPD at 6 months (ref. No)**								
Yes	0.05 (-0.11 to 0.21)	0.20* (0.01 to 0.39)	0.06 (-0.10 to 0.23)	0.23* (0.03 to 0.42)	0.09 (-0.07 to 0.26)	0.24* (0.04 to 0.44)	0.07 (-0.11 to 0.25)	0.25* (0.04 to 0.46)
**Maternal age (in years) (ref. ≤ 29)**								
30–34			0.05 (-0.20 to 0.30)	0.11 (-0.23 to 0.45)	-0.02 (-0.27 to 0.24)	0.10 (-0.25 to 0.44)	-0.03 (-0.31 to 0.25)	0.13 (-0.24 to 0.50)
35–39			0.10 (-0.14 to 0.34)	0.36* (0.03 to 0.68)	0.002 (-0.25 to 0.25)	0.30 (-0.04 to 0.64)	-0.06 (-0.33 to 0.21)	0.27 (-0.10 to 0.63)
≥ 40			0.01 (-0.24 to 0.26)	0.21 (-0.11 to 0.57)	-0.07 (-0.33 to 0.19)	0.17 (-0.16 to 0.55)	-0.16 (-0.45 to 0.12)	0.20 (-0.18 to 0.58)
**Maternal education (ref. high school or less)**								
CT/ VS or JC			0.23 (-0.20 to 0.48)	0.06 (-0.27 to 0.39)	0.30* (0.05 to 0.55)	0.09 (-0.25 to 0.42)	0.31* (0.03 to 0.58)	0.08 (-0.28 to 0.44)
University			0.27* (0.03 to 0.52)	0.16 (-0.17 to 0.49)	0.36** (0.12 to 0.61)	0.19 (-0.14 to 0.52)	0.37* (0.10 to 0.64)	0.19 (-0.16 to 0.55)
**Marital status (ref. single)**								
Married			0.08 (-0.45 to 0.62)	0.26 (-0.45 to 0.97)	0.06 (-0.49 to 0.62)	0.26 (-0.44 to 0.97)	0.02 (-0.54 to 0.58)	0.14 (-0.56 to 0.83)
**Occupation (ref. unemployed)**								
Employed			-0.01 (-0.13 to 0.11)	0.10 (-0.04 to 0.24)	-0.003 (-0.13 to 0.12)	0.10 (-0.05 to 0.25)	0.02 (-0.11 to 0.15)	0.09 (-0.06 to 0.25)
**Income (ref. < 4 million yen)**								
4–8 million			0.08 (-0.15 to 0.32)	0.08 (-0.22 to 0.38)	0.10 (-0.14 to 0.33)	0.07 (-0.23 to 0.38)	0.07 (-0.18 to 0.32)	-0.02 (-0.33 to 0.29)
> 8 million			0.03 (-0.13 to 0.11)	0.10 (-0.20 to 0.40)	0.05 (-0.18 to 0.29)	0.10 (-0.21 to 0.40)	0.05 (-0.20 to 0.30)	0.05 (-0.26 to 0.36)
**Child’s sex (ref. female)**								
Male			0.10 (-0.01 to 0.22)	-0.02 (-0.16 to 0.12)	0.10 (-0.02 to 0.21)	-0.03 (-0.17 to 0.12)	0.09 (-0.03 to 0.22)	-0.03 (-0.18 to 0.12)
**Gestational week (ref. Preterm < 37 weeks)**								
Full term ≥ 37 weeks			-0.22 (-0.51 to 0.07)	-0.48** (-0.82 to -0.13)	-0.07 (-0.37 to 0.22)	-0.40* (-0.75 to -0.04)	-0.09 (-0.40 to 0.23)	-0.22 (-0.51 to 0.07)
**Birth weight (in grams) (ref. < 2500)**								
≥ 2500			0.31** (0.11 to 0.52)	0.46*** (0.20 to 0.72)	0.27* (0.06 to 0.48)	0.43*** (0.17 to 0.69)	0.22 (0.01 to 0.44)	0.31* (0.04 to 0.58)
**Complications (ref. No)**								
Yes					-0.01 (-0.15 to 0.14)	-0.02 (-0.20 to 0.16)	0.004 (-0.15 to 0.16)	0.02 (-0.17 to 0.20)
**Delivery method (ref. Vaginal Delivery)**								
CS section					0.04 (-0.10 to 0.17)	-0.03 (-0.19 to 0.13)	-0.002 (-0.15 to 0.14)	-0.08 (-0.25 to 0.09)
**Pre pregnancy BMI (in kg/m**^**2**^**) (ref. 18.5 to 25)**								
< 18.5					0.28*** (-0.43 to -0.13)	-0.21* (-0.39 to -0.02)	-0.28** (-0.45 to -0.12)	-0.25 * (-0.44 to -0.05)
≥ 25					0.27* (0.20 to 0.52)	0.17 (-0.13 to 0.47)	0.23 (-0.04 to 0.49)	0.04 (-0.27to 0.36)
**Primiparity (ref. Yes)**								
No					0.01 (-0.12 to 0.13)	0.01 (-0.14 to 0.16)	0.04 (-0.09 to 0.18)	0.01 (-0.15 to 0.17)
**Exclusive breastfeeding until 6 months (ref. No)**								
Yes							-0.18* (-0.32 to -0.05)	-0.22* (-0.38 to -0.06)
**Initiation of Weaning Food at 6 months (ref. No)**								
Yes							0.07 (-0.06 to 0.20)	0.18* (0.03 to 0.34)

*CI* Confidence Interval, *BMI* Body Mass Index, *PPD* Postpartum depression, *CT or VS or JC* College of technology or vocational school or junior college

^*^P-value < 0.05, ^**^p-value < 0.01, ^***^p value < 0.001

We also considered the infant feeding practices as a potential mediator of the association between PPD at 6 months and child’s BMI z-score at 3 years. Based on the results of the primary associations observed in the multivariable analysis applied to the criteria described by Baron and Kenny, evidence of mediation was not observed in our study. However, PPD and infant feeding practices were independently associated with child’s BMI z-score at 3 years. Regarding potential effect modification, the statistical interaction between PPD at 6 months and exclusive breastfeeding until 6 months were tested and was not found to be statistically significant (*p* value 0.09).

Furthermore, we also performed sensitivity analysis by including overall population (not excluding multiple births) however, the results remain unchanged (Supplementary Table 2).

## Discussion

The current study examined the association between PPD and BMI z-score of the child at 1 and 3 years of age and assessed the role of infant feeding practices in influencing this association. We found that PPD at 6 months postpartum was positively associated with child’s BMI z-score at 3 years of age. Examination of infant feeding practices did not appear to mediate or modify the effect of PPD on child’s BMI z-score at 3 years. Characteristics such as maternal age, maternal education, pre-pregnancy BMI, birth weight of the child and infant feeding practices appeared to be independently associated with child’s BMI z-score.

In the context of the assessment for the timing of PPD, Suzumiya et al. (2004) in their Japanese study showed the prevalence of PPD at one month postpartum to be 1 to 2 times higher compared to other postpartum periods [32] which was consistent with our study with the prevalence of PPD at 1 month being higher than that of PPD at 6 months postpartum. In a U.S. study conducted among 1,359 women, the prevalence of PPD was about 34% in the first 6 months postpartum which is comparable to the results of studies conducted outside Japan [35]. In this study, we found an association between PPD at 6 months and child’s BMI z-score at 3 years of age. However, we did not find any association between PPD at 1 month and child’s BMI z-score at 1 and 3 years. The reasons behind this result might be the high rate of infant and mother health checkups 1 month after childbirth (83.6%) [36] and father’s child care leave up to 8 weeks postpartum in Japan [37] which may have helped mothers to receive care and support. As a result, the effect of PPD at 1 month may not be distinct within this period. However, with the increase in the employment rate of women in Japan, the burden might have increased for the PPD mothers which may compromise care-giving activities and performance of parenting roles affecting child’s growth and development [15, 16]. The association between PPD at 6 months and child’s BMI z-score at 3 years of age found is consistent with the result of a Brazilian cohort study showing an increased risk of overweight in infants aged 6-months to two years by 1.7 times among mothers with depressive symptoms compared to those without depressive symptoms [11]. In contrast, some studies performed in the USA and UK have shown PPD to be associated with slower growth of the infants [38, 39].

In this study, we also found that children who were exclusively breastfed had lower BMI z-score at 3 years of age, independent of the effect of PPD at 6 months. George et al. concluded that mothers with depressive symptoms were prone to have a less healthy lifestyle, and different feeding practices were provided to their infants [40]. Mothers with depressive symptoms were more likely to stop breastfeeding earlier than mothers who were non-depressed [41] which can be explained by the evidence from previous studies that the decrease in breastfeeding rates among depressed mothers may affect the child’s weight status [42–44]. The role of breastfeeding has been positively associated with child’s adequate growth and development, but studies have not consistently shown a relationship between exclusive breastfeeding and weight of child [45]. Thus, mother’s postpartum depression was speculated to affect negatively children feeding practices, especially for children less than 2 years of age. In a cross-sectional study conducted in nine Greek rural and urban regions, children who were exclusively breastfed showed a significantly lower prevalence of overweight and obesity at the age of 2–5 years [43]. This might suggest that exclusively breastfeeding an infant will help to maintain a healthy body weight as they grow.

Additionally, children who are not exclusively breastfed were reported to be at higher risk of being introduced to complementary feeding before the age of six months [46]. The findings of the Infant Feeding Practices Study II found an association of PPD with the early introduction to solid foods which had negative effect on child’s developmental outcomes [45]. Also, a study done examining the role of complementary foods on childhood obesity revealed that children who initiated weaning food before the age of four months and after six months of age were more likely to be overweight and obese in their later childhood [47]. This was also consistent with the current study as child’s BMI z-score was higher if their weaning was started before and after six months of their life.

In addition to PPD at 6 months and infant feeding practices, we were able to confirm the independent effect of other factors that influence higher BMI trajectories such younger gestational age, higher birthweight, and higher pre-pregnancy BMI. A study conducted in Poland reported a relationship between higher birth weight and higher BMI percentile of children aged 4–15 years [48]. A Chinese study showed the odds of childhood overweight to be significantly higher in children born to mothers who were overweight/obesity during the pre-pregnancy period than in those born to mothers with normal weight [49]. Similarly, findings from a longitudinal study showed an association between pre-pregnancy BMI and the obesity in children aged 2 years [50]. The findings from these studies were consistent with the results of our study suggesting that mothers should take good care of their diet and nutrition even before pregnancy. Taken together, the results of our multivariable analyses confirm the multi-factorial nature of early life factors in contributing to the weight status of children, of which our study has confirmed the role of PPD at 6 months, infant feeding practices, and several characteristics of the mother and child.

The availability of pregnancy-related factors, birth characteristics, psychosocial status, and longitudinal follow-up data in this cohort study provided the unique opportunity to perform one of the first studies of its kind in a Japanese population. The prospective design allowed us to minimize potential recall bias issues, such as those related to feeding practices. However, despite these strengths, there are important limitations to acknowledge. As with most longitudinal studies, there was loss to follow-up of the children over the 3 years leading to a reduced sample size which may have weakened our ability to examine potential interaction effects. Loss to follow-up was balanced between the PPD categories and characteristics of these participants were not markedly different from those who remained in the study. Self-reported data from mothers may have weaken the accuracy due to general reporting bias, we would expect this bias to be non-differential in nature and effect results towards the null. Finally, despite the high-quality data from this cohort study, this was a single center study with study subjects primarily residing in the same region of Tokyo. Thus, generalizability of the study findings may not be extendable into other sub-populations with vastly different characteristics. Additional studies are necessary to confirm these study findings in a wider range of populations. Nevertheless, results of our study highlight the importance of considering PPD as factor which should be considered in future preventative efforts.

## Conclusions

The study examined the association of PPD and infant feeding practices on relative weight of child at 1 year and 3 years of age. We found a significant association between PPD at 6 months postpartum and child’s BMI z-score at 3 years of age with infant feeding practices, maternal age, maternal education, pre-pregnancy BMI, birth weight of the child as likely confounders. Infant feeding practices did not appear to mediate or modify the association, but was found to be independently associated with child’s BMI z-score at 3 years. The significant relation of PPD at 6 months to child’s BMI z-score at 3 years, in conjunction with birth trends and high prevalence of PPD, can add to the body of evidence that there is need for multiple assessment across the first postpartum year to rule out PPD since early screening and early interventions may benefit both maternal health and child development outcomes. These findings can indicate the need for establishing support systems for appropriate mental health services, and also suggestive of health promotion initiatives providing support to continue breastfeeding practices for recommended duration and in care-giving activities for PPD mothers.

### Supplementary Information

**Supplementary Material 1.**

**Supplementary Material 2.**

## Data Availability

The datasets generated during and analyzed during the current study are not publicly available as it is a cohort data maintained by the National Center for Child Health and Development, Japan but are available from the institute itself on reasonable request. Authors Naho Morisaki and Hisako Tanaka could be contacted regarding any information on data sharing and availability.

## Abbreviations

US: United States
IRB: International Review Board
UK: United Kingdom
CI: Confidence Interval
SE: Standard Error
