# Supplementary material for: Effect of postpartum depression and role of infant feeding practices on relative weight of child at 1 and 3 years of age
Source: BMC Pregnancy Childbirth. 2024 May 2;24:336. doi: 10.1186/s12884-024-06483-2 (PMC11067203; doi:10.1186/s12884-024-06483-2)
Supplement: Supplementary file 1 — Supplementary Material 1. [file 12884_2024_6483_MOESM1_ESM.docx]

Supplementary Table 1: Characteristics of the study population stratified by PPD

|  | Overall Population  (n= 1038) | Population with PPD  (n=308) | Population without PPD  (n= 730) | P value |
| --- | --- | --- | --- | --- |
|  | **Frequency (%)** | **Frequency (%)** | **Frequency (%)** |  |
| Maternal age |  |  |  |  |
| <=29 | 64 (6.2) | 21 (6.8) | 43 (5.9) | 0.24 |
| 30-34 | 260 (25.1) | 89 (28.9) | 171 (23.4) |  |
| 35-39 | 483 (46.5) | 133 (43.2) | 350 (47.9) |  |
| >=40 | 231 (22.3) | 65 (21.1) | 166 (22.7) |  |
| Maternal education |  |  |  |  |
| High school or less | 63 (6.4) | 23 (7.5) | 40 (5.5) | 0.47 |
| CT or VS or JC | 324 (31.2) | 97 (31.5) | 227 (31.1) |  |
| University | 605 (58.3) | 176 (57.1) | 429 (58.8) |  |
| Missing | 46 (4.4) | 12 (3.9) | 34 (4.7) |  |
| Marital status |  |  |  |  |
| Single | 10 (1.0) | 2 (0.6) | 8 (1.1) | 0.5 |
| Married | 982 (94.6) | 293 (95.1) | 689 (94.4) |  |
| Missing | 46 (4.4) | 13 (4.2) | 33 (4.5) |  |
| Annual income (yen) |  |  |  |  |
| < 4 million | 74 (7.1) | 31 (10.1) | 43 (5.9) | 0.01* |
| 4-8 million | 331 (31.9) | 109 (35.4) | 222 (30.4) |  |
| > 8 million | 546 (52.6) | 143 (46.4) | 403 (55.2) |  |
| Missing | 87 (8.4) | 25 (8.1) | 62 (8.5) |  |
| Occupation |  |  |  |  |
| Unemployed | 435 (41.9) | 116 (37.7) | 319 (43.7) | 0.07 |
| Pre-pregnancy BMI (kg/m^2^) | |  |  |  |
| 18.5-25 | 765 (73.7) | 216 (70.1) | 549 (75.2) | 0.06 |
| <18.5 | 199 (19.2) | 63 (20.5) | 136 (18.6) |  |
| >=25 | 62 (6.0) | 26 (8.4) | 36 (4.9) |  |
| Missing | 12 (1.2) | 3 (1.0) | 9 (1.2) |  |
| Complications during pregnancy ^c^ | |  |  |  |
| No | 800 (77.1) | 238 (77.3) | 562 (77.0) | 0.92 |
| Yes | 238 (22.9) | 70 (22.7) | 168 (23.0) |  |
| Delivery method |  |  |  |  |
| Vaginal delivery | 703 (67.7) | 213 (69.2) | 490 (67.1) | 0.5 |
| CS section | 302 (29.1) | 85 (27.6) | 217 (29.7) |  |
| Missing | 33 (3.2) | 10 (3.2) | 23 (3.2) |  |
| Primiparity |  |  |  |  |
| Yes | 666 (64.2) | 222 (72.1) | 444 (60.8) | 0.001** |
| No | 372 (35.8) | 86 (27.9) | 286 (39.2) |  |
| Gender |  |  |  |  |
| Male | 517 (49.8) | 161 (52.3) | 356 (48.8) | 0.3 |
| Female | 521 (50.2) | 147 (47.7) | 374 (51.2) |  |
| Gestational week |  |  |  |  |
| Preterm <37 weeks | 53 (5.1) | 17 (5.5) | 36 (4.9) | 0.7 |
| Fullterm ≥ 37 weeks | 984 (94.8) | 291 (94.5) | 693 (94.9) |  |
| Missing | 1 (0.1) |  | 1 (0.1) |  |
| Birth weight (in grams) |  |  |  |  |
| Low (<2500) | 112 (10.8) | 33 (10.7) | 79 (10.8) | 0.9 |
| Normal (2500-4000) | 918 (88.4) | 272 (88.3) | 646 (88.5) |  |
| Obese (≥ 4000) | 8 (0.8) | 3 (1.0) | 5 (0.7) |  |

PPD- Postpartum depression, CT or VS or JC : College of technology or vocational school or junior college, ^c^ – presence of at least one of the following complications: gestational diabetes mellitus, pregnancy-induced hypertension, pre-eclampsia, multiple pregnancy, placenta accreta, and placenta previa, *- p-value<0.05, **- p-value<0.01, ***- p value<0.001
