# Supplementary material for: Effect of postpartum depression and role of infant feeding practices on relative weight of child at 1 and 3 years of age
Source: BMC Pregnancy Childbirth. 2024 May 2;24:336. doi: 10.1186/s12884-024-06483-2 (PMC11067203; doi:10.1186/s12884-024-06483-2)
Supplement: Supplementary file 2 — Supplementary Material 2. [file 12884_2024_6483_MOESM2_ESM.docx]

Supplementary Table 2: Multivariable linear regression model examining the association between PPD and children's BMI z-score

|  | **Model 1** | |  | **Model 2** | |
| --- | --- | --- | --- | --- | --- |
|  | **1 year**  **(N =832)** | **3 years**  **(N =620)** |  | **1 year**  **(N =847)** | **3 years**  **(N =637)** |
| **PPD at 1 month (ref. No)** | |  | **PPD at 6 months (ref. No)** | |  |
| Yes | -0.03 (-0.19 to 0.13) | 0.15 (-0.04 to 0.35) | Yes | 0.09 (-0.08 to 0.26) | 0.25 (0.06 to 0.45) * |
|  |  |  |  |  |  |
| **Maternal age (in years) (ref. ≤ 29)** | | | **Maternal age (in years) (ref. ≤ 29)** | | |
| 30-34 | -0.01 (-0.28 to 0.25) | 0.26 (-0.08 to 0.60) | 30-34 | -0.01 (-0.27 to 0.25) | 0.25 (-0.10 to 0.59) |
| 35-39 | -0.03 (-0.28 to 0.23) | 0.41 (0.08 to 0.74) * | 35-39 | -0.04 (-0.29 to 0.21) | 0.39 (0.05 to 0.72) * |
| ≥ 40 | -0.08 (-0.36 to 0.19) | 0.39 (-0.05 to 0.73) * | ≥ 40 | -0.12 (-0.39 to 0.15) | 0.33 (-0.01 to 0.67) |
| **Maternal education (ref. high school or less)** | |  | **Maternal education (ref. high school or less)** | |  |
| CT or VS or JC | 0.31 (0.03 to 0.59) * | 0.13 (-0.22 to 0.48) | CT or VS or JC | 0.25 (-0.02 to 0.51) | -0.002 (-0.34 to 0.33) |
| University | 0.32 (0.04 to 0.59) * | 0.23 (-0.11 to 0.58) | University | 0.35 (0.09 to 0.61) * | 0.14 (-0.19 to 0.47) |
| **Marital status (ref. single)** | |  | **Marital status (ref. single)** | |  |
| Married | -0.01 (-0.60 to 0.60) | 0.36 (-0.39 to 1.11) | Married | 0.02 (-0.54 to 0.58) | 0.14 (-0.55 to 0.82) |
| **Occupation (ref. unemployed)** | |  | **Occupation (ref. unemployed)** | |  |
| Employed | 0.05 (-0.09 to 0.18) | 0.07 (-0.08 to 0.23) | Employed | 0.01 (-0.12 to 0.14) | 0.07 (-0.08 to 0.22) |
| **Income (ref. < 4 million yen)** | |  | **Income (ref. < 4 million yen)** | |  |
| 4-8 million | -0.01 (-0.26 to 0.23) | -0.10 (-0.40 to 0.20) | 4-8 million | 0.04 (-0.20 to 0.27) | -0.06 (-0.35 to 0.23) |
| > 8 million | -0.04 (-0.29 to 0.20) | -0.05 (-0.35 to 0.25) | > 8 million | 0.01 (-0.22 to 0.25) | 0.01 (-0.28 to 0.30) |
| **Child’s sex (ref. female)** | |  | **Child’s sex (ref. female)** | |  |
| Male | 0.10 (-0.03 to 0.22) | 0.03 (-0.12 to 0.17) | Male | 0.08 (-0.05 to 0.20) | -0.02 (-0.16 to 0.12) |
| **Gestational week (ref. Preterm < 37 weeks)** | |  | **Gestational week (ref. Preterm < 37 weeks)** | |  |
| Full term ≥ 37 weeks | -0.15 (-0.45 to 0.15) | -0.36 (-0.71 to 0.02) * | Full term ≥ 37 weeks | -0.11 (-0.38 to 0.16) | -0.51 (-0.82 to -0.20) ** |
| **Birth weight (in grams) (ref. < 2500)** | |  | **Birth weight (in grams) (ref. < 2500)** | |  |
| ≥ 2500 | 0.25 (0.03 to 0.47) * | 0.36 (0.1 to 0.67) ** | ≥ 2500 | 0.18 (0.03 to 0.38) | 0.26 (0.01 to 0.51) * |
| **Complications (ref. No)** | |  | **Complications (ref. No)** | |  |
| Yes | 0.02 (-0.14 to 0.18) | 0.03 (-0.16 to 0.22) | Yes | 0.001 (-0.15 to 0.16) | 0.001 (-0.18 to 0.18) |
| **Delivery method (ref. Vaginal Delivery)** | |  | **Delivery method (ref. Vaginal Delivery)** | |  |
| CS section | 0.001 (-0.14 to 0.15) | -0.10 (-0.26 to 0.07) | CS section | -0.01 (-0.15 to 0.13) | -0.09 (-0.25 to 0.08) |
| **Pre pregnancy BMI (in kg/m^2^) (ref. 18.5 to 25)** | | | **Pre pregnancy BMI (in kg/m^2^) (ref. 18.5 to 25)** | | |
| < 18.5 | -0.33 (-0.49 to -0.17) *** | -0.28 (-0.50 to -0.09) ** | < 18.5 | -0.25 (-0.41 to -0.09) * | -0.21 (-0.40 to -0.03) * |
| ≥ 25 | 0.25 (-0.02 to 0.51) | 0.11 (-0.20 to 0.43) | ≥ 25 | 0.25 (-0.01 to 0.51) | 0.09 (-0.21 to 0.40) |
| **Primiparity (ref. Yes)** | |  | **Primiparity (ref. Yes)** | |  |
| No | 0.06 (-0.08 to 0.19) | 0.02 (-0.14 to 0.18) | No | 0.05 (-0.08 to 0.18) | 0.03 (-0.12 to 0.19) |
| **Multiple pregnancy (ref. No)** | |  | **Multiple pregnancy (ref. No)** | |  |
| Yes | -0.003 (-0.41 to 0.41) | 0.16 (-0.33 to 0.65) | Yes | 0.10 (-0.20 to 0.41) | 0.01 (-0.36 to 0.39) |
| **Exclusive breastfeeding until 6 months (ref. No)** | |  | **Exclusive breastfeeding until 6 months (ref. No)** | |  |
| Yes | -0.15 (-0.33 to -0.02) * | -0.18 (-0.34 to -0.02) * | Yes | -0.18 (-0.32 to -0.05) * | -0.22 (-0.36 to -0.05) * |
| **Initiation of Weaning Food at 6 months (ref. No)** | |  | **Initiation of Weaning Food at 6 months (ref. No)** | |  |
| Yes | 0.05 (-0.08 to 0.17) | 0.16 (0.003 to 0.31) * | Yes | 0.06 (-0.07 to 0.18) | 0.17 (0.02 to 0.32) * |
|  |  |  |  |  |  |

Model 1- Multivariable linear regression model showing association between PPD at one month and child’s BMI at 1 year and 3 year adjusted for the sociodemographic factors, pregnancy related factors, child factors, and multiple pregnancy, Model 2- Multivariable linear regression model showing association between PPD at six months and child’s BMI at 1 year and 3 year adjusted for the sociodemographic factors, pregnancy related factors, child factors, and multiple pregnancy, CI- Confidence Interval, BMI- Body Mass Index, PPD- Postpartum depression, CT or VS or JC : College of technology or vocational school or junior college, *- p-value<0.05, **- p-value<0.01, ***- p value<0.001
